# Supplementary material for: Cretaceous Connections Among Camel Cricket Lineages in the Himalaya Revealed Through Fossil-Calibrated Mitogenomic Phylogenetics
Source: Insects. 2025 Jun 27;16(7):670. doi: 10.3390/insects16070670 (PMC12294936; doi:10.3390/insects16070670)
Supplement: Supplementary file 1 [file insects-16-00670-s001.zip › insects-3659252-supplementary.pdf]

Table S1. Rhaphidophorid (117-taxa) included in this study with location details and GenBank accession numbers. Approximately 3,314 bp DNA sequence data consists of 2071 mitochondrial DNA (COI=1197, 12S=368 and 16S=504) and 1233 Nuclear rRNA (551 18S and 682 28S). Additional taxa newly sequenced for this study are in bold. Two representatives of Prophalangopsidae (*Cyphoderris monstrosa* and *Tarragoilus diuturnus*) were employed as an outgroup.

Sub Family	Taxa	Code	Location	GeneBank accession number					Reference
				COI	12S	16S	18S	28S	
Aemodogryllinae	*Diestramima tsongkhapa*	MPN_CW5525	Bhutan	OR896622 (complete mt genome)					**This study**
	Diestramima matermagna	MPN_CW5536	Bhutan	OR896621 (complete mt genome)					**This study**
	D. acutiapicis	DI037GZ	Lianhuaping, Leigong Mountain, Guizhou, China	MW961044	MW962019	MW961784	MW961666	MW961902	Zhu et al. 2022
	D. arbora	DI068GX	Shiwanda Mountain, Shangsi, Guangxi, China	MW961064	MW962039	MW961804	MW961686	MW961922	Zhu et al. 2022
	D. austrosinensis	DI033ZJ	Pan'an County, Jinhua City, Zhejiang, China	MW961042	MW962017	MW961782	MW961664	MW961900	Zhu et al. 2022
	D. beybienkoi	DI004GZ	Mayang River, Tongren City, Guizhou, China	MW961025	MW962000	MW961765	MW961647	MW961883	Zhu et al. 2022
	D. bina	DI792HN	Mang Mountain, Yizhang County, Hunan, China	MW961119	MW962091	MW961859	MW961741	MW961977	Zhu et al. 2022
	D. brevis	DI027ZJ	Tianmu Mountain, Zhejiang, China	MW961038	MW962013	MW961778	MW961725	MW961961	Zhu et al. 2022
	D.conica	DI060YN	Baihualing, Boshan City, Yunnan, China	MW961057	MW962032	MW961797	MW961679	MW961915	Zhu et al. 2022
	D. cryptopygia	DI062YN	Tongbiguan Village, Yingjiang County, Yunnan, China	MW961058	MW962033	MW961798	MW961680	MW961916	Zhu et al. 2022
	D. cycla	DI040SC	Wanniansi, Emei Mountain, Sichuan, China	MW961047	MW962022	MW961787	MW961669	MW961905	Zhu et al. 2022
	D. excavata	DI639GX	Huaping, Longsheng, Guangxi, China	MW961076	MW962048	MW961816	MW961698	MW961934	Zhu et al. 2022
	D. furcata	DI807GX	Baihe Napo, Guangxi, China	MW961132	MW962103	MW961872	MW961754	MW961989	Zhu et al. 2022
	D. guangxiensis	DI687GX	Daming Mountain, Nanning City, Guangxi, China	MW961096	MW962068	MW961836	MW961718	MW961954	Zhu et al. 2022
	D. hainanensis	DI631HN	Wuzhi Mountain, Hainan, China	MW961071	MW962043	MW961811	MW961693	MW961929	Zhu et al. 2022
	D. intermedia	DI001SC	Hongkou Village, Dujiangyan City, Sichuan, China	MW961024	MW961999	MW961764	MW961646	MW961882	Zhu et al. 2022

D. lamina	DI066GX	Nonggang, Longzhou County, Guangxi, China	MW961062	MW962037	MW961802	MW961684	MW961920	Zhu et al. 2022
D. longifolia	DI801YN	Maandi Village, Jinping, Yunnan, China	MW961128	MW962099	MW961868	MW961750	MW961985	Zhu et al. 2022
D. qionglaiensis	DI657SC	Nanmuxi, Qionglai City, Sichuan, China	MW961094	MW962066	MW961834	MW961716	MW961952	Zhu et al. 2022
D. sichuanensis	DI794SC	Huagaoxi, Xuyong County, Sichuan, China	MW961121	MW962093	MW961861	MW961743		Zhu et al. 2022
D. spinata	DI046YN	Gulinjing Village, Maguan County, Yunnan, China	MW961050	MW962025	MW961790	MW961672	MW961908	Zhu et al. 2022
D. subrectis	DI045GX	Dayao Mountain, Jiuxiu County, Guangxi, China	MW961049	MW962024	MW961789	MW961726	MW961962	Zhu et al. 2022
D. subtilis	DI029YN	Wenlong Village, Pu'er City, Yunnan, China	MW961040	MW962015	MW961780	MW961662	MW961898	Zhu et al. 2022
D. taiwanensis	DI083TW	Jilong City, Taiwan	MW961067	MW962042	MW961807	MW961689	MW961925	Zhu et al. 2022
D. tibetensis	DI070XZ	80K, Medog County, Xizang, China	MW961066	MW962041	MW961806	MW961688	MW961924	Zhu et al. 2022
D. triangulata	DI028GZ	Leigong Mountain, Guizhou, China	MW961039	MW962014	MW961779	MW961661	MW961897	Zhu et al. 2022
D. truncata	DI036GX	Dayao Mountain, Jiuxiu County, Guangxi, China	MW961043	MW962018	MW961783	MW961665	MW961901	Zhu et al. 2022
D. yunnanensis	DI809YN	Menglun Botanical Garden, Yunnan, China	MW961134	MW962105	MW961874	MW961756	MW961991	Zhu et al. 2022
Diestrammena sp.		China	MT849270					Yuan et al. 2020
D. asynamora		China	KX057726				KR903068	Zhou et al. 2017; Chintauan-Marquier et al. 2016
D. fengyangshanica	DD199ZJ	Zhejiang, Longquan, Fengyangshan, China	ON129823	ON146711	ON146820	ON146765	ON146655	Zhu et al 2022
D. japanica		Japan	MK347245					Guan, & Xu, 2018
Gymnaetoides deformus	PT127ZJ	Zhejiang, Linan, Tianmu Mountain, China	ON129846	ON146678	ON146788	ON146734	ON146622	Zhu et al. 2022
G. huangshanensis	GY879AH	Anhui, Huang Mountain, Tangkou, China	ON129833	ON146665	ON146775	ON146721	ON146609	Zhu et al. 2022
G. lushanensis	GY113JX	Jiangxi, Lu Mountain, Guling, China	ON129825	ON146657	ON146767	ON146713	ON146601	Zhu et al. 2022
G. petalus	GY112AH	Anhui, Chizhou City, Shitai County, Gongxi Village, Du hamlet, Penglaixian Cave, China	ON129824	ON146656	ON146766	ON146712	ON146600	Zhu et al. 2022

	G. testaceus	GY115ZJ	Zhejiang, Linan, Tianmu Mountain, China	ON129827	ON146659	ON146769	ON146715	ON146603	Zhu et al. 2022
	G. yangmingensis	GY733HN	Hunan, Shuangpai, Yangmingshan, China	ON129830	ON146662	ON146772	ON146718	ON146606	Zhu et al. 2022
	G. yueyangensis	PT120JX	Jiangxi, Lu Mountain, Guling, China	ON129841	ON146673	ON146783	ON146729	ON146617	Zhu et al. 2022
	Homotachycines acutilobatus	GY731HB	Hubei, Shennongjia, Huangbaiqian, China	ON129829	ON146661	ON146771	ON146717	ON146605	Zhu et al. 2022
	H. baokangensis	PT824HB	Hubei, Baokang, Houping, China	ON129864	ON146696	ON146806	ON146750	ON146640	Zhu et al. 2022
	H. concavus	PT830HN	Henan, Luoyang, Longyuwan, China	ON129867	ON146699	ON146809	ON146753	ON146643	Zhu et al. 2022
	H. fusus	PT663SX	Shaanxi, Yang County, Huayang, China	ON129849	ON146681	ON146791	ON146737	ON146625	Zhu et al. 2022
	H. ovalilobatus	PT746HB	Hubei, Shennongjia, Huangbaiqian, China	ON129858	ON146690	ON146800	ON146744	ON146634	Zhu et al. 2022
	H. qinlingensis	PT662SX	Shaanxi, Yang County, Huayang, China	ON129848	ON146680	ON146790	ON146736	ON146624	Zhu et al. 2022
	H. quadratus	PT124HB	Hubei, Shennongjia, Banbiyan, China	ON129845	ON146677	ON146787	ON146733	ON146621	Zhu et al. 2022
	H. triangulus	PT122HB	Hubei, Shennongjia, Maohu, China	ON129843	ON146675	ON146785	ON146731	ON146619	Zhu et al. 2022
	Pseudotachycines fengyangshanensis	PT744ZJ	Zhejiang, Longquan, Fengyangshan, China	ON129857	ON146689	ON146799	ON146743	ON146633	Zhu et al. 2022
	P. nephrus	PT742ZJ	Zhejiang, Linan, Qingliangfeng, China	ON129855	ON146687	ON146797		ON146631	Zhu et al. 2022
	P. procerus	PT822AH	Anhui, Yuexi, Yaoluoping, China	ON129862	ON146694	ON146804	ON146748	ON146638	Zhu et al. 2022
	P. procerus guizhouensis	PT110GZ	Guizhou, Suiyang, Kuankuoshui, China	ON129837	ON146669	ON146779	ON146725	ON146613	Zhu et al. 2022
	P. sagittus	PT667YN	Yunnan, Yiliang, Chaotianma, China	ON129850	ON146682	ON146792	ON146738	ON146626	Zhu et al. 2022
	P. volutus	PT108ZJ	Zhejiang, Linan, Tianmu Mountain, China	ON129835	ON146667	ON146777	ON146723	ON146611	Zhu et al. 2022
	P. zhengi	PT118JX	Jiangxi, Lu Mountain, Guling, China	ON129839	ON146671	ON146781	ON146727	ON146615	Zhu et al. 2022
	Tachycines shuangcha		China		OM993275.1				Hang 2020
	T. zorzini	MW322826	China	NC_057442					Y. Wang et al. 2021
Ceuthophilinae	*Ceuthophilus* sp.	MPN_CW4347	Moab Desert, USA	OR880641 (complete mt genome)					This study
	C. carlsbadensis			KU377020	Z97597	Z97613	Z97563		Flook et al. 1999; Weckstein et al. 2016

	C. gracilipes	CEU/GRA	Hamden, CT, USA	AY793593	MK993663	AY793561		MK993657	Allegrucci et al. 2019; Allegrucci et al. 2005
	Euhadenoecus insolitus	INDe	Indian Grave Point Cave, The Kalb Co., TN, USA	AY793591	F216948	AY793563	MK993679	EF217005	Allegrucci et al. 2019; Allegrucci et al. 2005
	Hadenoecus cumberlandicus	BATh	at Cave, Carter Cave State Park, Carter Co., KY, USA	AY793592	EF216947	AY793562	MK993680	EF217004	Allegrucci et al. 2019; Allegrucci et al. 2005
Dolichopodainae	*Dolichopoda annae*	TEM	Aghlia Paraskevi Cave, Tembi Valley, Larissa, Greece	EU887894	EU887846	EU887861	MK993723	EU887875	Allegrucci et al. 2019; Allegrucci et al. 2009
	D. araneiformis	VEL	Velika Cave, Blato, Miljet, Croatia	EF217019	EF216944	EF216974.1	MK993720	EF216982	Allegrucci et al. 2019; Allegrucci et al. 2009
	D. cassagnaui	TRI	Aghlia Triada Cave, Karistos, Eubea Island, Greece	EF217035	EF216931	EF216961	MK993737	EF216991	Allegrucci et al. 2019; Allegrucci et al. 2009
	D. dalensi	KEF	Kefalovrisi Cave, Argos, Argolide, Peloponnesus	EF217026	EF216929	EF216959	MK993745	EF216987	Allegrucci et al. 2019; Allegrucci et al. 2009
	D. gasparoi	CHI	Chirospilia Cave, Evghiros, Levkada, Ionian Islands	EF217008	EF216920	EF216950	MK993727	EF216976	Allegrucci et al. 2019; Allegrucci et al. 2009
	D. giachinoi	ORO	Megalospilio Cave, Monastirakion, Aitolo-Akarnania, Greece	EF217012	EF216922	EF216952	MK993731	EF216978	Allegrucci et al. 2019; Allegrucci et al. 2009
	D. graeca	PER	Perama Cave, Ioannina, Epiro, Greece	EF217013	EF216923	EF216953	MK993725	EF216979	Allegrucci et al. 2019; Allegrucci et al. 2009
	D. hussoni	IZB	Apano Skala Cave, Naoussa, Imathia, Greece	EF217031	EF216943	EF216973	MK993722	EF216990	Allegrucci et al. 2019; Allegrucci et al. 2009
	D. insignis	PAN	Panos Cave, Marathon, Athene, Attica, Greece	EF217054.1	EF216938	EF216968	MK993735	EF217000	Allegrucci et al. 2019; Allegrucci et al. 2009
	D. ithakii	ITA	Marmarospilia cave, Vathi, Ithaki Island, Ionian Islands	EF217006	EF216919	EF216949	MK993728	EF216975	Allegrucci et al. 2019; Allegrucci et al. 2009
	D. kiriakii	AGH	Kiriaki Cave, Korifè, Aghlia Kiriaki, Parga, Greece	EF217014	EF216924	EF216954	MK993732	EF216980	Allegrucci et al. 2019; Allegrucci et al. 2009
	D. linderi	SIR	Sirach Cave Eastern Pyrenees, France	AY793598	JF826039	AY793567	MK993708	JF826061	Allegrucci et al. 2019; Allegrucci et al. 2005
	D. lustriae	AND	Aghios Andreas Cave, Valtou M., Halkiopuli, Etolia, Greece	EU887901.1	EU887848	EU887863	MK993733	EU887878	Allegrucci et al. 2019; Allegrucci et al. 2009
	D. makrykapa	PKI	Paralia Kilidau Cave, Lamari, Eubea Island, Greece	EF217042.1	EF216941	EF216971	MK993739	EF216993	Allegrucci et al. 2019; Allegrucci et al. 2009
	D. matsakisi	ANA	Analipsi Cave, Pititsa, Achaia, Peloponnesus	EF217022	EF216927	EF216957	MK993744	EF216985	Allegrucci et al. 2019; Allegrucci et al. 2009

	D. naxia	ZEU	Zeus Cave, Filotas, Naxos Island, Cyklades, Greece	EU887909	EU887853	EU887868	MK993741	EU887882	Allegrucci et al. 2019; Allegrucci et al. 2009
	D. paraskevi	PAR	Aghlia Paraskevi Cave, Skotinon, Iraklio, Crete Island	EF217027	EF216942	EF216972.1	MK993747		Allegrucci et al. 2019; Allegrucci et al. 2009
	D. patrizii	PET	Small cave, Petalas, Ionian Islands	EU887898	EU887847	EU887862	MK993729	EU887877	Allegrucci et al. 2019; Allegrucci et al. 2009
	D. pavesii	SPI	Drogarati Cave, Sami, Kefalonia Island, Ionian Islands	EF217010	EF216921	EF216951	MK993730	EF216977	Allegrucci et al. 2019; Allegrucci et al. 2009
	D. petrochilosi	JOA	Aghlia Joannis, Nea Pendeli, Athene, Attica, Greece	EF217053	EF216937	EF216967	MK993736	EF216999	Allegrucci et al. 2019; Allegrucci et al. 2009
	D. remyi	POZ	Pozarska Mala Pestera, Loutrakiou, Pella, Greece	AY793637	EF216939	EF216969	MK993721	EF217001	Allegrucci et al. 2019; Allegrucci et al. 2005
	D. sbordonii	KAR	Karain Cave, Antalya, Turkey	EF217050	EF216936	EF216966	MK993748	EF216998	Allegrucci et al. 2019; Allegrucci et al. 2009
	D. steriotisi	ANT	Antropograva Cave, Klimatia, Kerkira, Corfu, Ionian Islands	EF217016	EF216925	EF216955	MK993726	EF216981	Allegrucci et al. 2019; Allegrucci et al. 2009
	D. thasosensis	DRA	Drakotripa Cave, Panayia, Thasos Island, Kavala, Greece	EF217020	EF216926	EF216956	MK993724	EF216983	Allegrucci et al. 2019; Allegrucci et al. 2009
	D. unicolor	KAT	Kataphingi Cave, Selitsa, Messenia, Peloponnesus	EF217045	EF216940	EF216970	MK993746	EF216994	Allegrucci et al. 2019; Allegrucci et al. 2009
	D. vandeli	HER	Hermes Cave, Orkomenos, Dhionisos, Beotia, Greece	EF217039	EF216932	EF216962	MK993734	EF216992	Allegrucci et al. 2019; Allegrucci et al. 2009
Rhaphidophorinae	*Rhaphidophora bicuspis*	MPN_ CW5529	Bhutan	OR896623 (complete mt genome)					This study
	R. quadrispina		China	OL450400					Lu, X 2021 (retrieved from GenBank)
	R. bilobata	MPN_CW5545	Bhutan	OR896626 (complete mt genome)					This study
	R. bhutanensis	MPN_CW5483	Bhutan	OR896625 (complete mt genome)					This study
	Stonychophora sp.	MPN_ORT15	Bethel, Solomon Is.	OR896624 (complete mt genome)					This study
Troglophilinae	*Troglophilus adamovici*	A4E	Konya, Seydisehir, Tinatzepe cave, Turkey	KY412419.1	KY412232	KY412279	KY412325	KY412372	Allegrucci et al. 2017
	T. alanyaensis	T22	Alanya, Dim Cave, Turkey	KY412414.1	KY412227	KY412274	KY412320	KY412367	Allegrucci et al. 2017
	T. andreinii	ANA	Putignano, Fico cave, Italy	KY412385.1	KY412198	KY412245	KY412292	KY412338	Allegrucci et al. 2017
	T. bicakcii	I1N	Konya, Derebucak,Bicacki cave, Turkey	KY412427.1	KY412240	KY412287	KY412333	KY412380	Allegrucci et al. 2017

	T. brevicauda	T9	Skrapar, Pirro cave, Albania	KY412398.1	KY412211	KY412256.1	KY412305	KY412351	Allegrucci et al. 2017
	T. cavicola	CAV	Bergamo, Grone, Belon cave, Italy	KY412388	KY412201.1	KY412248.1	MK993751	EF217002	Allegrucci et al. 2017
	T. escalerai	A10	Karaman- Maras, Dongel cave, Turkey	KY412418.1	KY412231	KY412278	KY412324	KY412371	Allegrucci et al. 2017
	T. ferzenensis	ADA2B	Konya, Ferzene cave, Turkey	KY412422.1	KY412235	KY412282	KY412328	KY412375	Allegrucci et al. 2017
	T. fethiyiensis	E1D	Mugla Fethiye, Guroluk cave	KY412424.1	KY412237	KY412284	KY412330	KY412377	Allegrucci et al. 2017
	T. gajaci	A9E	Icel Silifke, Cennet cave, Turkey	KY412421.1	KY412234	KY412281	KY412327	KY412374	Allegrucci et al. 2017
	T. lagoi	LAG	Rhodos Island, Greece	KY412407.1	KY412220	KY412267	KY412313	KY412360	Allegrucci et al. 2017
	T. lazaropolensis	T31	Macedonia, Lazaropole, Macedonia	KY412394.1	KY412207	KY412254	KY412301	KY412347	Allegrucci et al. 2017
	T. marinae	SAN	Santorini Island, Greece	KY412408.1	KY412221	KY412268	KY412314	KY412361	Allegrucci et al. 2017
	T. neglectus	NEG	Sagrado, Proteo cave, Italy	EU938374			KF570820	KF570948	Fenn et al. 2008; Allegrucci et al. 2017
	T. ovuliformis	T32	Zavala Vjetrenica, Herzegovina	KY412395	KY412208	KY412255	KY412302	KY412348	Allegrucci et al. 2017
	T. ozeli	E5D	Havran cave, Turkey	KY412426.1	KY412239	KY412286	KY412332	KY412379	Allegrucci et al. 2017
	T. spinulosus	SPI	Crete, 11.29 km northeast of Chania, near Gouverneto, Cave Spilaio Lera (Stavros), 184 m, Greece	KY412412.1	KY412225	KY412272	KY412318	KY412365	Allegrucci et al. 2017
	T. tatyanae	KAF	Artvin, Kafkasor, Turkey	KY412431.1	KY412244	KY412291	KY412337	KY412384	Allegrucci et al. 2017
	T. zoiai	MAY	Fokidos (Mt. Giona), Stromi, Mayer's Cave, Greece	KY412402.1	KY412216	KY412263	KY412310	KY412356	Allegrucci et al. 2017
Cyphoderrinae	*Cyphoderris monstrosa*			KM657332					Song et al. 2015
Prophalangopsinae	*Tarragoilus diuturnus*			NC_021397					Zhou et al. 2014

Note: *Diestrammena fengyangshanica* is described in student thesis which is publicly unavailable and also it is not included in the Orthoptera species list.

Table S2. The best-fitting partitioning scheme for phylogenetic analyses and divergence time estimation; 1) using 13 mitochondrial coding genes and 2) short sequences (117-taxa set).

	Partition method	BEST Substitution model	Subset partition	Alignment	Site model in BEAST	Non-standard substitution model implemented in BEAUti
1	13 Coding Genes	GTR+F+I+G4	atp6_co1_co2_co3_cytb_nd3	1-675 832-2373 2374-3042 3043-3828 3829-4962 6901-7251	GTR	
		GTR+F+I+G4	atp8_nd2_nd6	676-831 5872-6900 10615-11136	GTR	
		TIM+F+R4	nd1_nd4_nd4L_nd5	4963-5871 7252-8589 8590-8883 8884-10614	GTR	Transition model, AC=GT, AT=CG and unequal base freq.
2	Gene and codon position	GTR+F+R5	co1	1-1197;		
		TIM3e+I+G4	co1_pos1	1-1197		
		TN+F+R2	co1_pos2	2-1197		
		TPM2u+F+G4	co1_pos3	3-1197		
		GTR+F+I+I+R4	co1_16s	1198-1566 1567-2077		
		TIM2e+I+I+R2	co1_28s	2078-2628 2629-3314		

Table S3. Mitochondrial genome annotation table of newly mapped sample sequences from North America, Bhutan, and Solomon Island. Asterix (*) represent incomplete TAA stop codon in the mRNA.

mt genome composition		CW5536 *Diestramima* sp1	CW5525 *Diestramima tsongkhapa*	CW5529 *Rhaphidophora* sp. 1	CW5483 Rhaphidophorinae gen. 2 sp. 1	CW5545 Rhaphidophorinae gen. 3 sp. 1	CW4347 *Ceuthophilus* sp.	ORT015_Rhaphidophorinae_gen. 1 sp. 1	Direction
rRNA	12S rRNA	789	789	821	793	791	787	792	reverse
	16S rRNA	1359	1333	1364	1362	1359	1325	1239	reverse
CDS	ATP6 CDS	678	678	678	678	678	678	678	forward
	ATP8 CDS	159	159	159	159	159	159	150	forward
	COX1 CDS	1545	1545	1545	1545	1545	1545	1545	forward
	COX2 CDS	691*	691*	690	690	690	688	688	forward
	COX3 CDS	789	789	789	789	789	789	789	forward
	CYTB CDS	1137	1137	1137	1137	1137	1137	1137	forward

	ND1 CDS	951	951	951	951	951	951	951	reverse
	ND2 CDS	1029	1029	1029	1032	1029	1029	1029	forward
	ND3 CDS	354	354	354	354	354	354	354	forward
	ND4 CDS	1339*	1339*	1339*	1339*	1339*	1333*	1341	reverse
	ND4L CDS	294	294	294	294	294	294	294	reverse
	ND5 CDS	1737	1737	1732*	1732	1732*	1732*	1734	reverse
	ND6 CDS	528	528	528	528	528	519	528	forward
Total coding genes length		11231	11231	11225	11228	11225	11208	11218	
tRNA	tRNA-Ala	63	63	63	64	63	65	64	forward
	tRNA-Arg	64	64	65	66	65	62	64	forward
	tRNA-Asn	67	67	67	67	67	64	66	forward
	tRNA-Asp	66	66	66	68	66	66	69	forward
	tRNA-Cys	66	66	71	66	67	66	60	reverse
	tRNA-Gln	69	69	69	74	69	67	69	reverse
	tRNA-Glu	68	68	68	69	68	64	66	forward
	tRNA-Gly	67	67	66	67	67	61	67	forward
	tRNA-His	66	66	63	65	64	62	68	reverse
	tRNA-Ile	65	65	66	66	66	65	70	forward
	tRNA-Leu	67	67	66	67	67	68	66	forward
	tRNA-Leu	65	65	65	67	66	65	64	reverse
	tRNA-Lys	70	69	69	70	69	69	71	forward
	tRNA-Met	69	69	69	71	70	68	69	forward
	tRNA-Phe	66	66	66	66	67	63	66	reverse
	tRNA-Pro	67	67	66	66	66	68	69	reverse
	tRNA-Ser	70	70	69	70	69	68	71	forward
	tRNA-Ser	68	68	67	67	67	66	70	forward
	tRNA-Thr	64	64	67	69	70	67	68	forward
	tRNA-Trp	68	68	69	66	68	68	71	forward

tRNA-Tyr	68	68	66	66	67	66	50	reverse
tRNA-Val	71	71	72	70	71	71	71	reverse
Total genome length	14853	14826	14885	14870	14854	14769	14718	

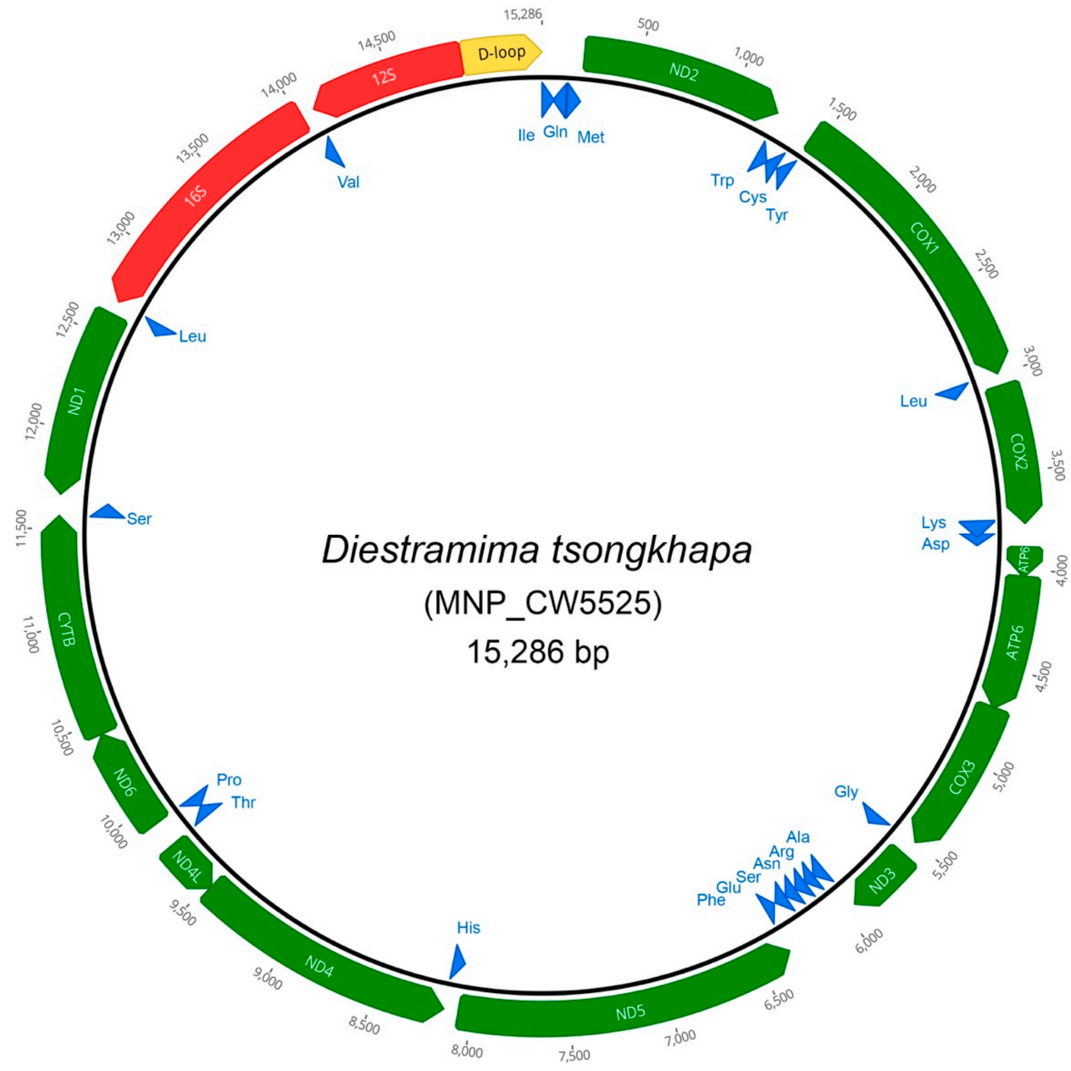

Figure S1. The mitochondrial genome of *Diestramima tsongkhapa* (MPN_CW5525) with 13 protein coding genes in green, 2 rRNAs in red, 22 tRNA genes in blue and A + T rich repeat region in yellow. The direction of each annotation is indicated with arrow.

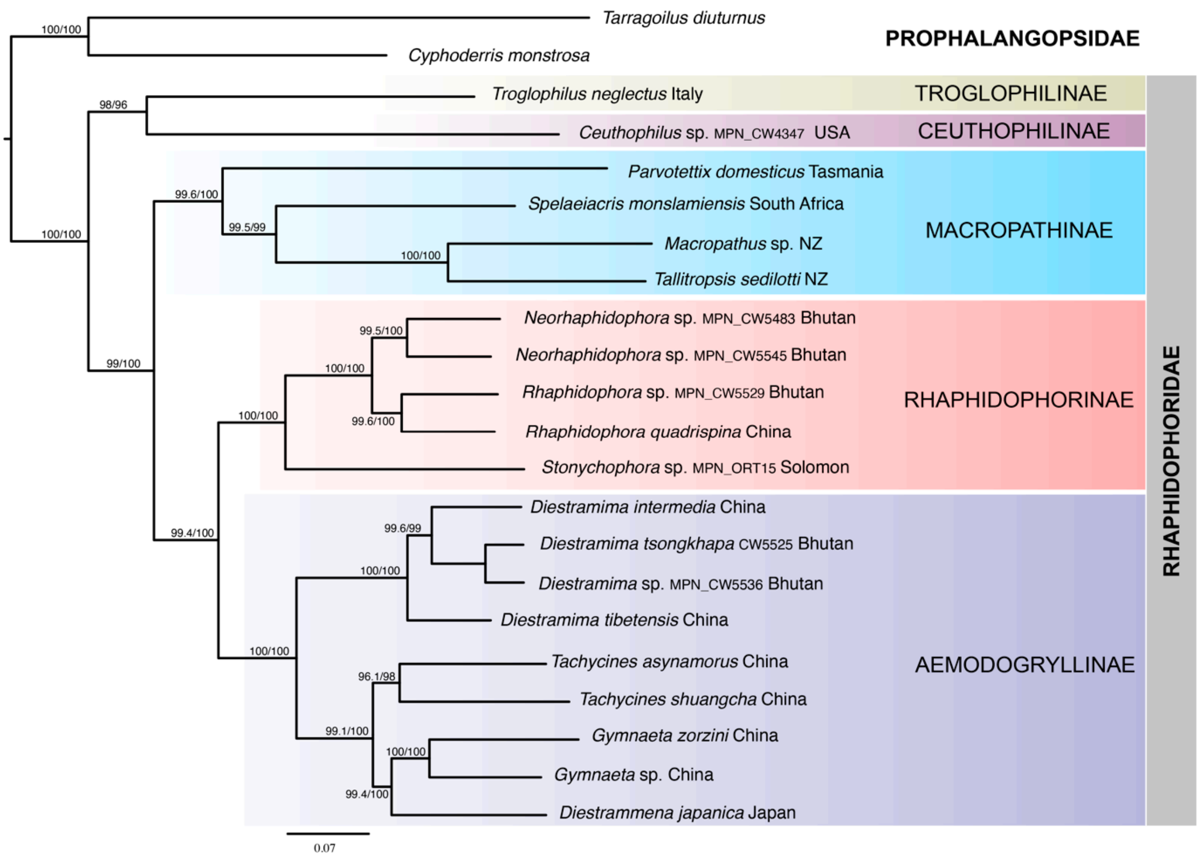

Figure S2. Evolutionary relationships of Rhaphidophoridae inferred from nucleotide alignment of 13 concatenated mitochondrial protein coding genes (11,124 bp) by ML analysis. Values on nodes are Sh-aLRT/bootstrap support values (1000 replicates).

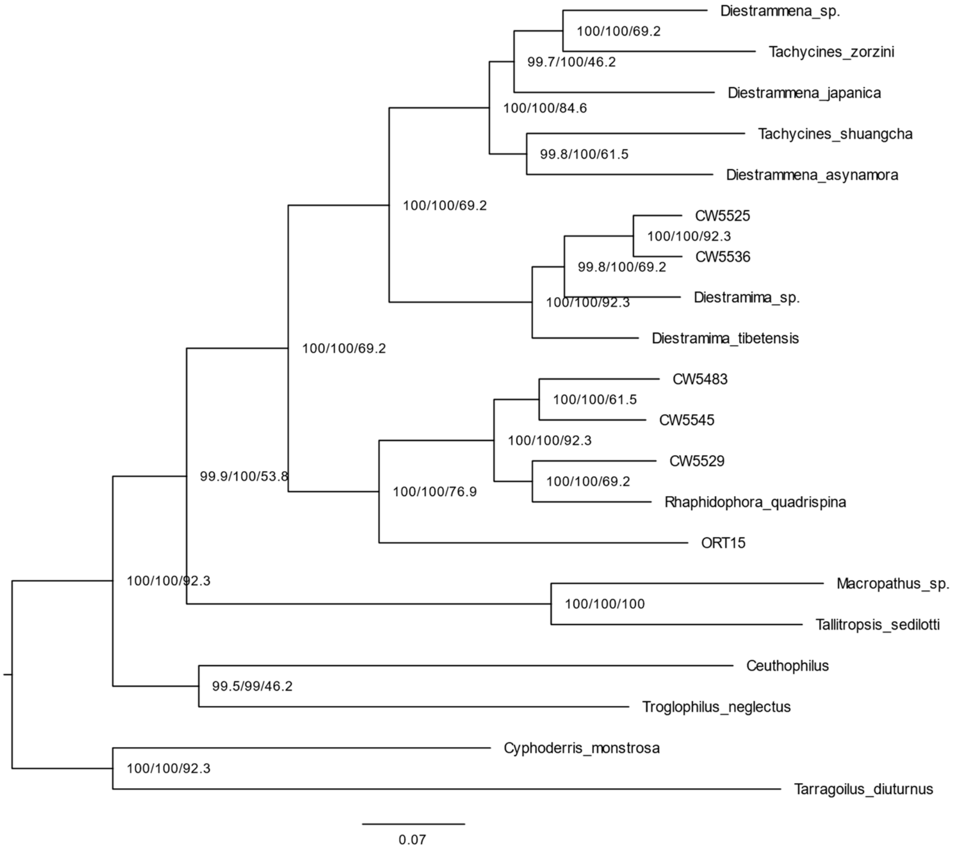

Figure S3. ML tree generated using IQ TREE. Gene concordance factor of complete mitochondrial coding genes of Rhaphidophoridae. The values on the nodes represent ultra-fast bootstrap values and SH-aLRT values of reference tree (Gene tree) and gene concordance factor.

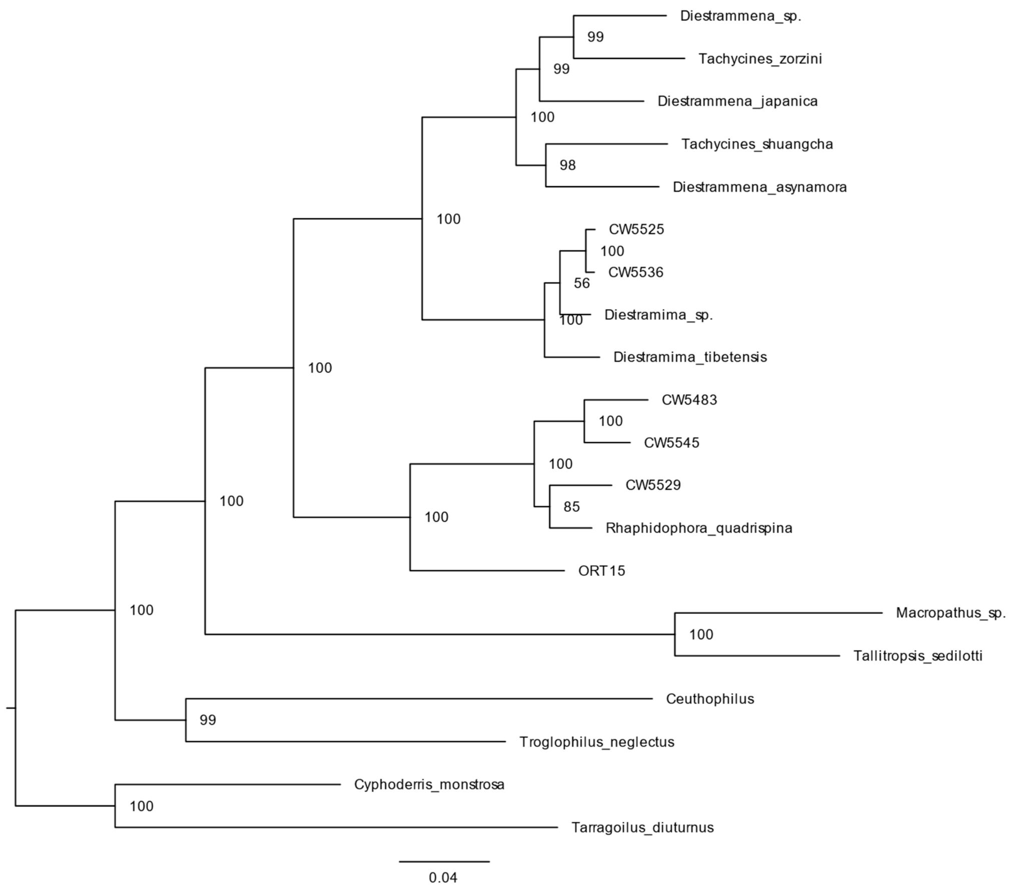

Figure S4. ML tree generated using IQ TREE. Phylogenetic tree constructed using the complete mitochondrial amino acid alignment sequence of Rhaphidophoridae. The values on nodes represent Ultra-fast bootstrap values.

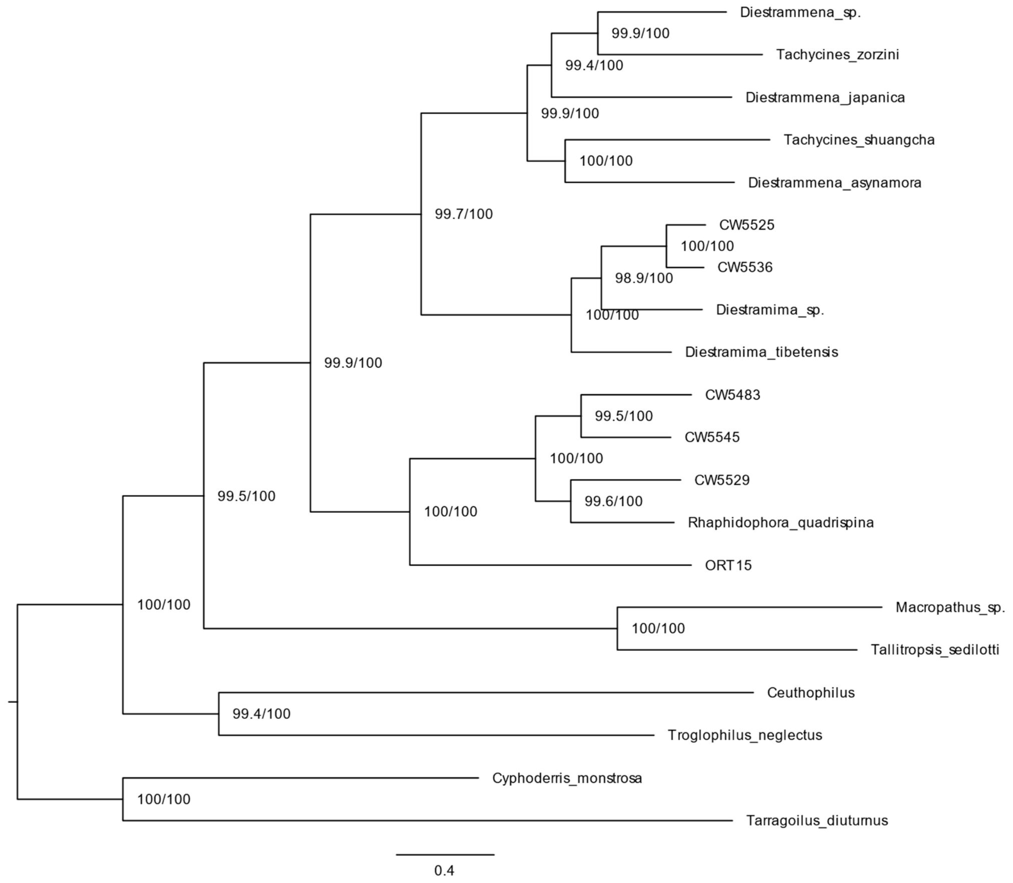

Figure S5. ML tree generated using IQ TREE. Complete mitochondrial DNA phylogeny of Rhaphidophoridae infer on 13 coding genes partition by gene and codon positions (52 partitions). The values on the nodes represent Ultra-fast bootstrap and SH-aLRT values.

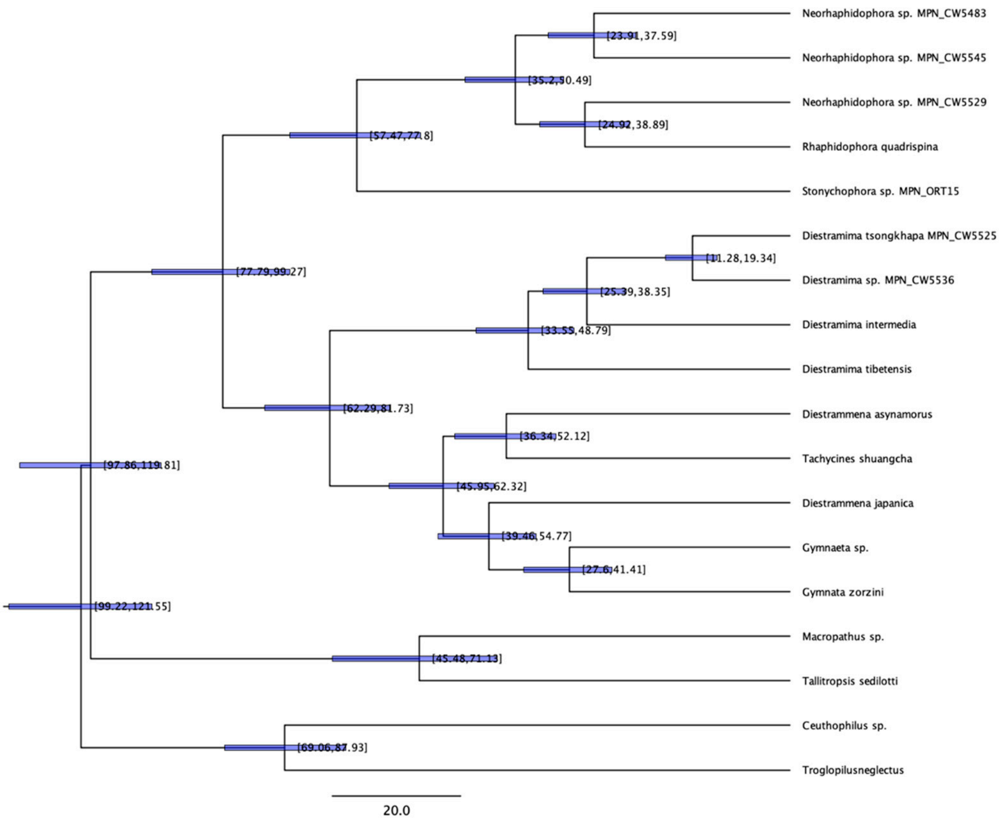

Figure S6. Chronogram of Rhaphidophoridae emphasising Asian lineages inferred by Bayesian analysis using points corresponding to divergent of Troglophilinae from Ceuthophilinae and Rhaphidophorinae and Aemodogryllinae from Macropathinae from previous analysis based on short sequences and secondary landscape-calibration (Allegrucci & Sbordoni, 2019). Node bars represent 95% Highest Posterior Density credibility intervals.
